# Supplementary material for: Hyperpolarized Dihydroxyacetone Is a Sensitive Probe of Hepatic Gluconeogenic State
Source: Metabolites. 2021 Jul 5;11(7):441. doi: 10.3390/metabo11070441 (PMC8307483; doi:10.3390/metabo11070441)
Supplement: Supplementary file 1 [file metabolites-11-00441-s001.zip › metabolites-1268792-supplementary.pdf]

Supporting information accompanying

Hyperpolarized Dihydroxyacetone is a Sensitive Probe of Hepatic Gluconeogenic State

Mukundan Ragavan[1,†], Marc A. McLeod[1,†], Anthony G. Giacalone[1], and Matthew E. Merritt[1,*]

[1]Department of Biochemistry and Molecular Biology, University of Florida, Gainesville, FL 32610, USA
mukundan@ufl.edu (M.R); marc.mcleod@ufl.edu (M.A.M); anthonygiacalone@ufl.edu (A.G.G)

*Correspondence: matthewmerritt@ufl.edu

† indicates equal contribution

List of Figures

Figure Number	Figure caption
S1	Endogenous glucose production over the entire period of perfusion (from the start of the perfusion to freeze clamp including HP DHA administration; ~ 34 minutes). Error bars are standard deviation. n = 6 (db/db), 5 (glucagon), and 6 (metformin). "*****" denotes statistical significance ($p < 0.05$).
S2	Ratio of signal intensities of six-carbon (glucose-6-phosphate and glucose – both α- and β- isomers) to three- and four-carbon (glyceraldehyde-3-phosphate, phosphoenol pyruvate, glycerol, glycerol-3-phosphate, lactate, and malate) resonances. Malate resonance (shown in Fig. 2) was not present in all spectra and was used for calculating 6C/3C ratio when present. Signal intensities were obtained by fitting a mixed Gaussian/Lorentzian shape to resonances in the sum spectra (shown in Fig. 2). * indicates statistical significance ($p < 0.05$). Error bars are standard deviation. n = 6 (db/db), 5 (glucagon), 6 (metformin), and 5 (control). Perfusate used in the 'Control' group did not contain propionate. Propionate concentrations in other groups are sufficiently low to not affect hepatic energetics and gluconeogenesis [1,2]
S3	Fractional enrichment of glucose C1-3 and C4-6 fragments in the perfusate. Glucose from the perfusate predominantly contains glucose produced prior to HP DHA injection and hence is representative of glucose produced from sources including glycogen and propionate. Error bars are standard deviation. n = 6 (*db/db*), 5 (glucagon), and 6 (metformin).
S4	Comparison of glucose enrichment in C1-3 fragment from perfusate and liver. Perfusate represents glucose produced prior to HP DHA injection and liver represents glucose produced post HP DHA injection. * represents statistical significance ($p < 0.05$, Student's T-test).
S5	Comparison of glucose enrichment in C4-6 fragment from perfusate and liver. Perfusate represents glucose produced prior to HP DHA injection and liver represents glucose produced post HP DHA injection. * represents statistical significance ($p < 0.05$, Student's T-test).
S6	Pool sizes of metabolites in the liver quantified using GC-MS. Inset showing metabolites other than lactate and alanine is included for clarity.
S7	Fractional enrichments of 3-carbon metabolites extracted from the liver as estimated using GC-MS. Error bars are standard deviation. n = 6 (db/db), 5 (glucagon), and 6 (metformin). ***** denotes statistical significance ($p < 0.05$).
S8	Fractional enrichments of citric acid cycle intermediates and closely associated metabolites extracted from the liver as estimated using GC-MS. Error bars are standard deviation. n = 6 (*db/db*), 5 (glucagon), and 6 (metformin). * denotes statistical significance ($p < 0.05$).

List of Tables

Table Number	Table Heading
S1	Metabolites identified post HP-DHA administration from ^{13}C spectra from perfused db/db, glucagon-treated, and metformin-treated livers. “Y” indicates peaks were observed in the ^{13}C spectra. Not all metabolites were observed in every spectrum.
S2	Metabolites derivatized by Methoxyamine hydrochloride and dimethyl tert butyl silyl trifluoroacetamide (MBSTFA) and their associated quantitation ions for GC-MS identification
S3	Glucose fragments generated after derivatization by hydroxylamine hydrochloride and propionic anhydride and their associated quantitation ions for GC-MS identification

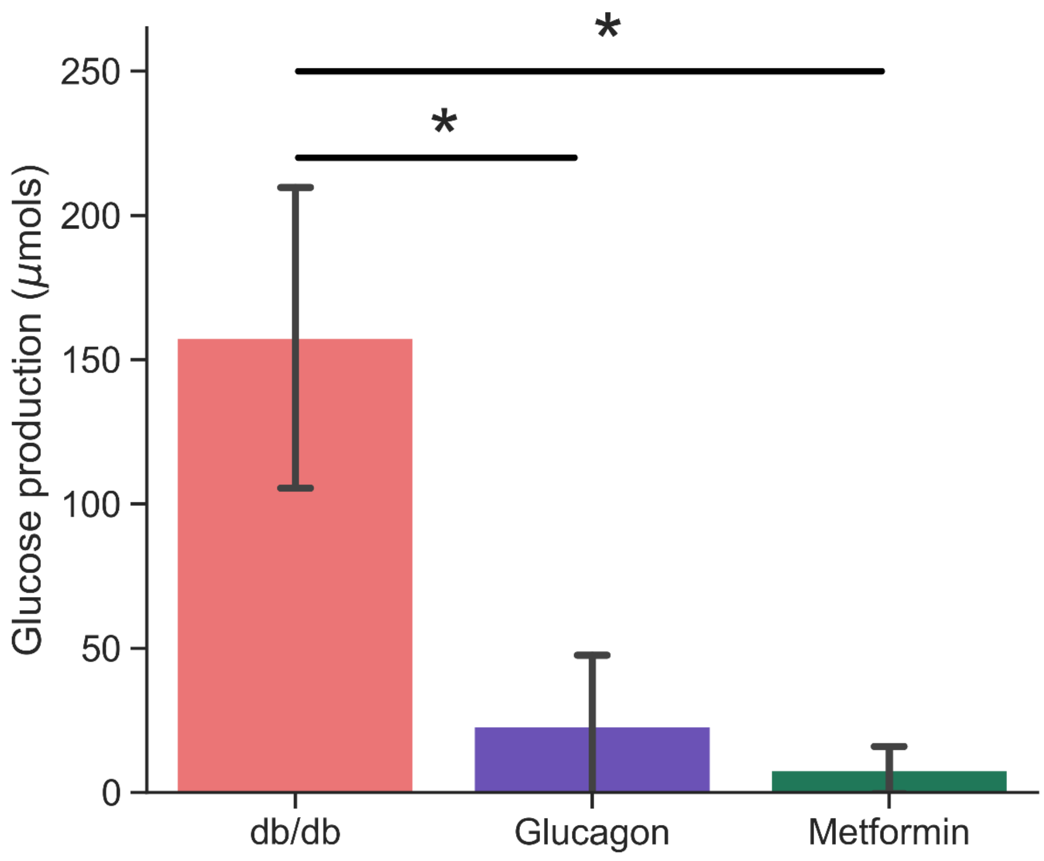

Figure S1. Endogenous glucose production over the entire period of perfusion (from the start of the perfusion to freeze clamp including HP DHA administration; ~ 34 minutes). Error bars are standard deviation. n = 6 (db/db), 5 (glucagon), and 6 (metformin). ***** denotes statistical significance ($p < 0.05$).

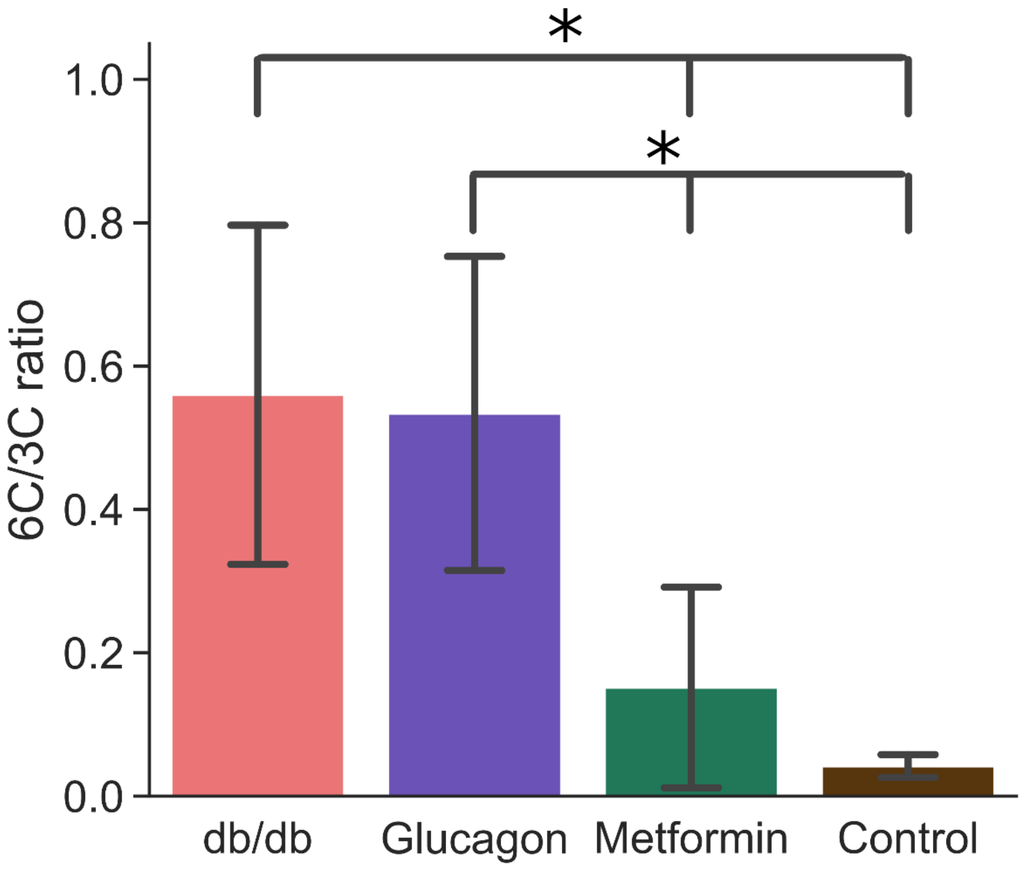

Figure S2. Ratio of signal intensities of six-carbon (glucose-6-phosphate and glucose – both α- and β-isomers) to three- and four-carbon (glyceraldehyde-3-phosphate, phosphoenol pyruvate, glycerol, glycerol-3-phosphate, lactate, and malate) resonances. Malate resonance (shown in Fig. 2) was not present in all spectra and was used for calculating 6C/3C ratio when present. Signal intensities were obtained by fitting a mixed Gaussian/Lorentzian shape to resonances in the sum spectra (shown in Fig. 2). * indicates statistical significance ($p < 0.05$). Error bars are standard deviation. n = 6 (db/db), 5 (glucagon), 6 (metformin), and 5 (control). Perfusate used in the 'Control' group did not contain propionate. Propionate concentrations in other groups are sufficiently low to not affect hepatic energetics and gluconeogenesis [1,2]

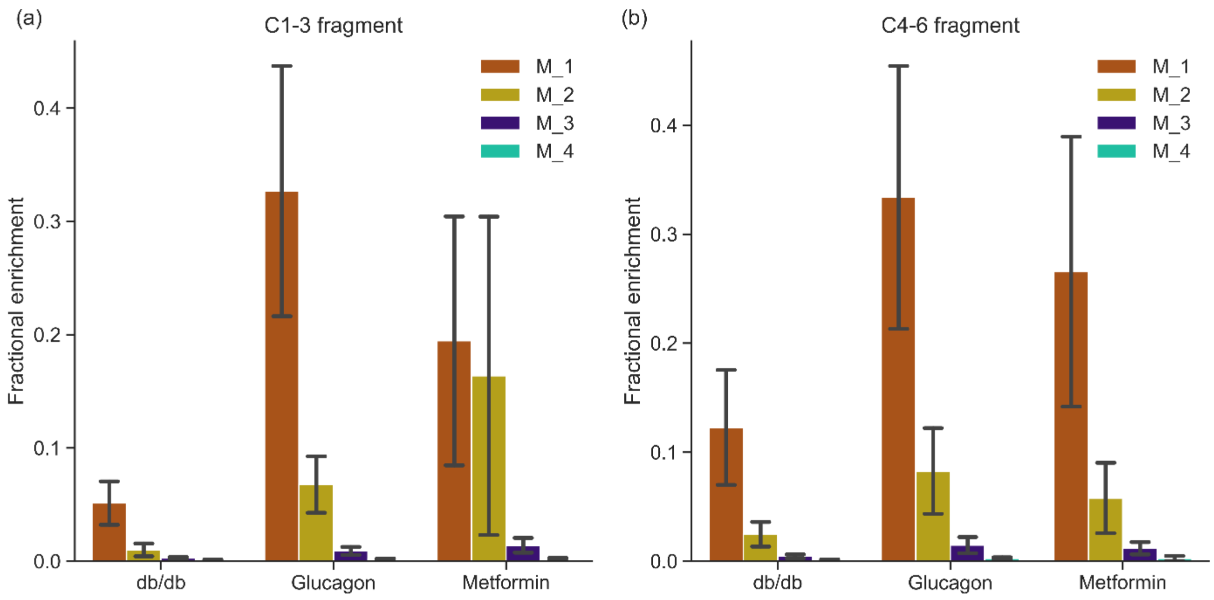

Figure S3. Fractional enrichment of glucose C1-3 and C4-6 fragments in the perfusate. Glucose from the perfusate predominantly contains glucose produced prior to HP DHA injection and hence is representative of glucose produced from sources including glycogen and propionate. Error bars are standard deviation. n = 6 (*db/db*), 5 (glucagon), and 6 (metformin).

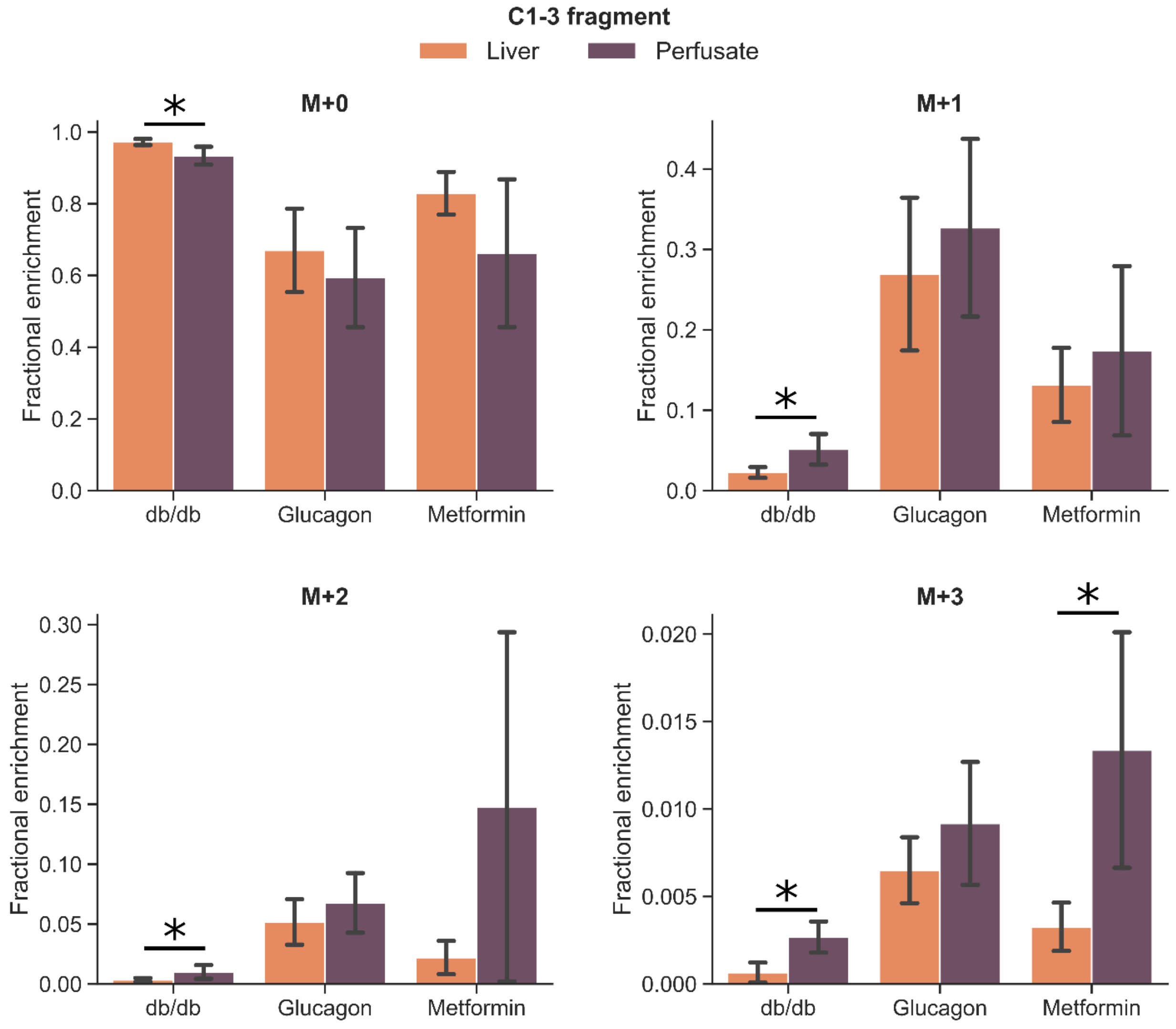

Figure S4. Comparison of glucose enrichment in C1-3 fragment from perfusate and liver. Perfusate represents glucose produced prior to HP DHA injection and liver represents glucose produced post HP DHA injection. * represents statistical significance ($p < 0.05$, Student's T-test).

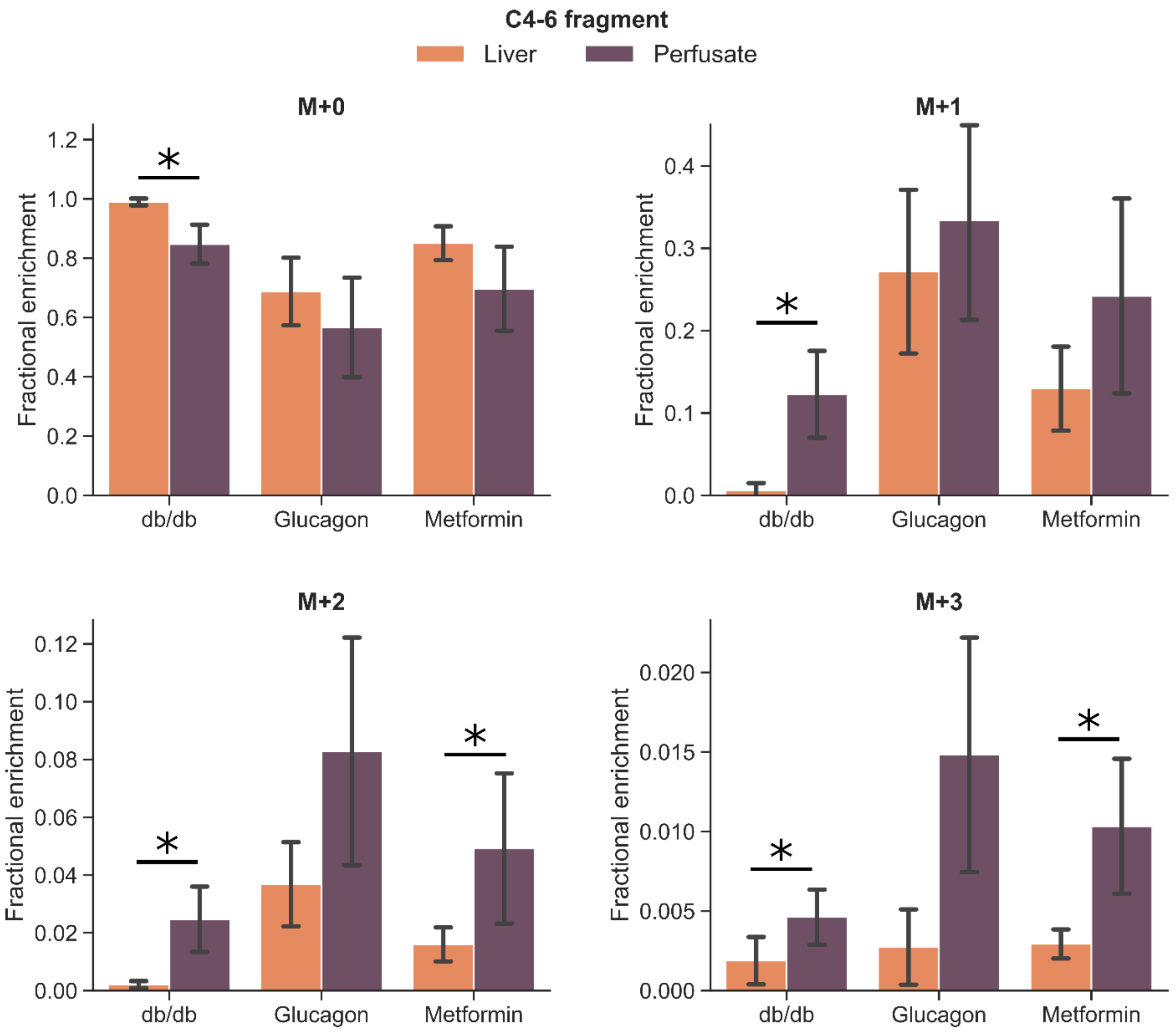

Figure S5. Comparison of glucose enrichment in C4-6 fragment from perfusate and liver. Perfusate represents glucose produced prior to HP DHA injection and liver represents glucose produced post HP DHA injection. * represents statistical significance ($p < 0.05$, Student's T-test).

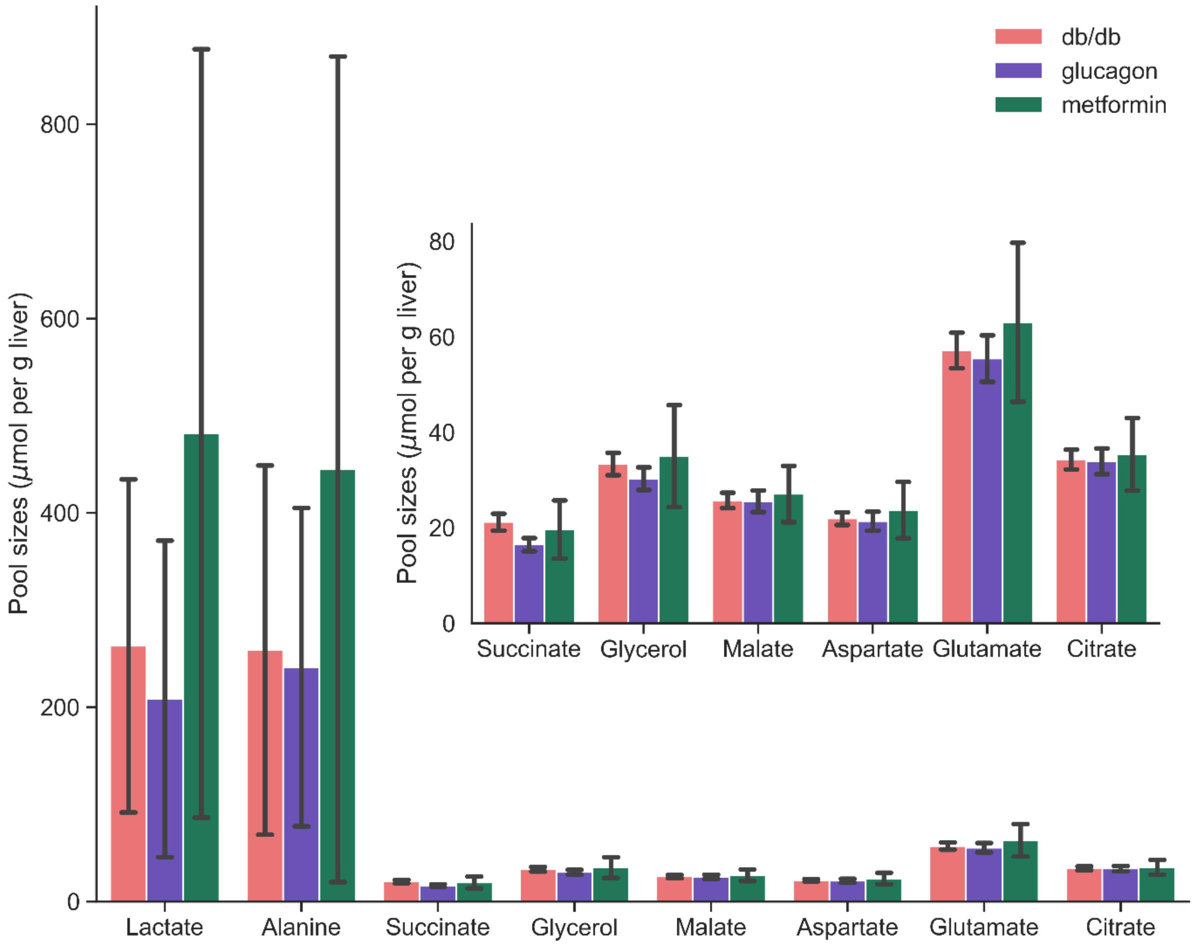

Figure S6. Pool sizes of metabolites in the liver quantified using GC-MS. Inset showing metabolites other than lactate and alanine is included for clarity.

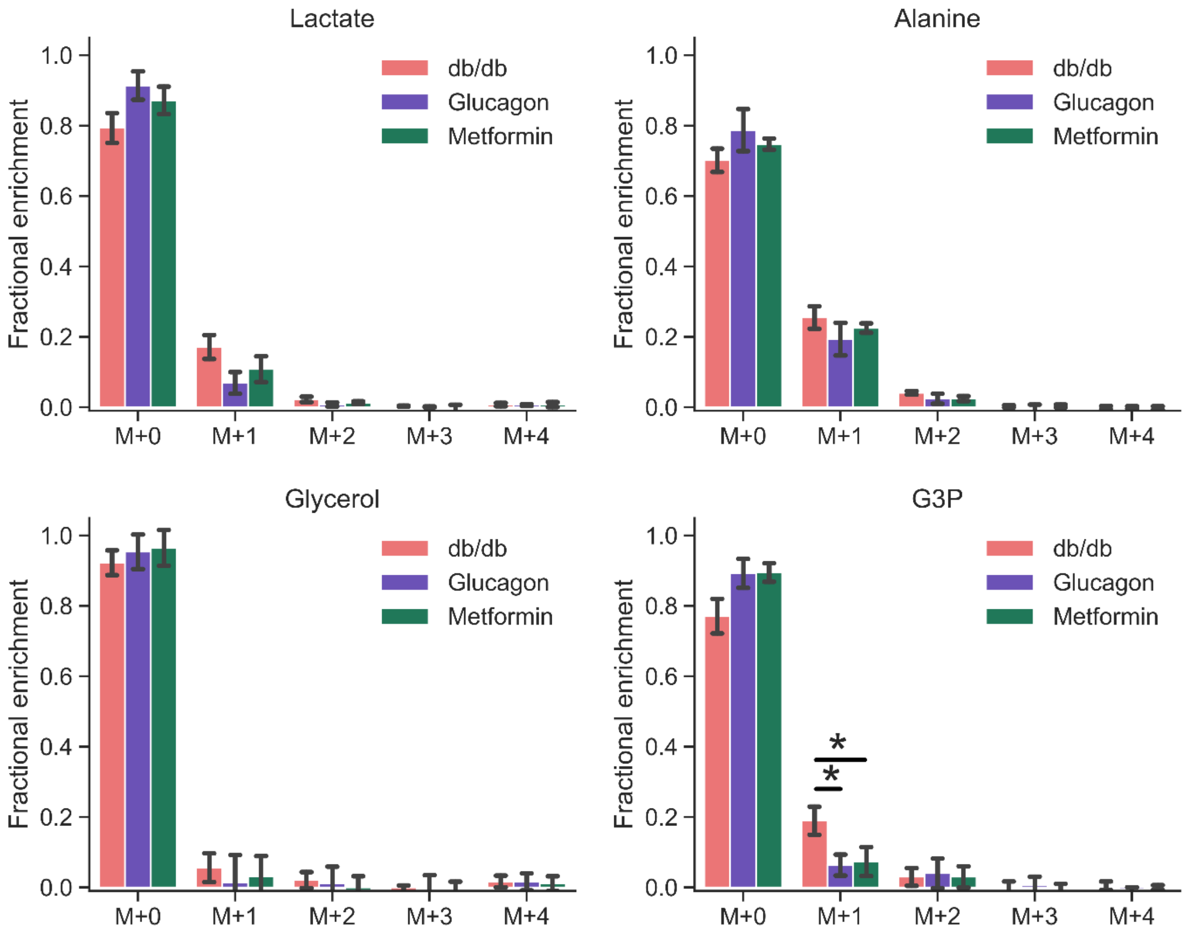

Figure S7. Fractional enrichments of 3-carbon metabolites extracted from the liver as estimated using GC-MS. Error bars are standard deviation. n = 6 (db/db), 5 (glucagon), and 6 (metformin). * denotes statistical significance ($p < 0.05$).

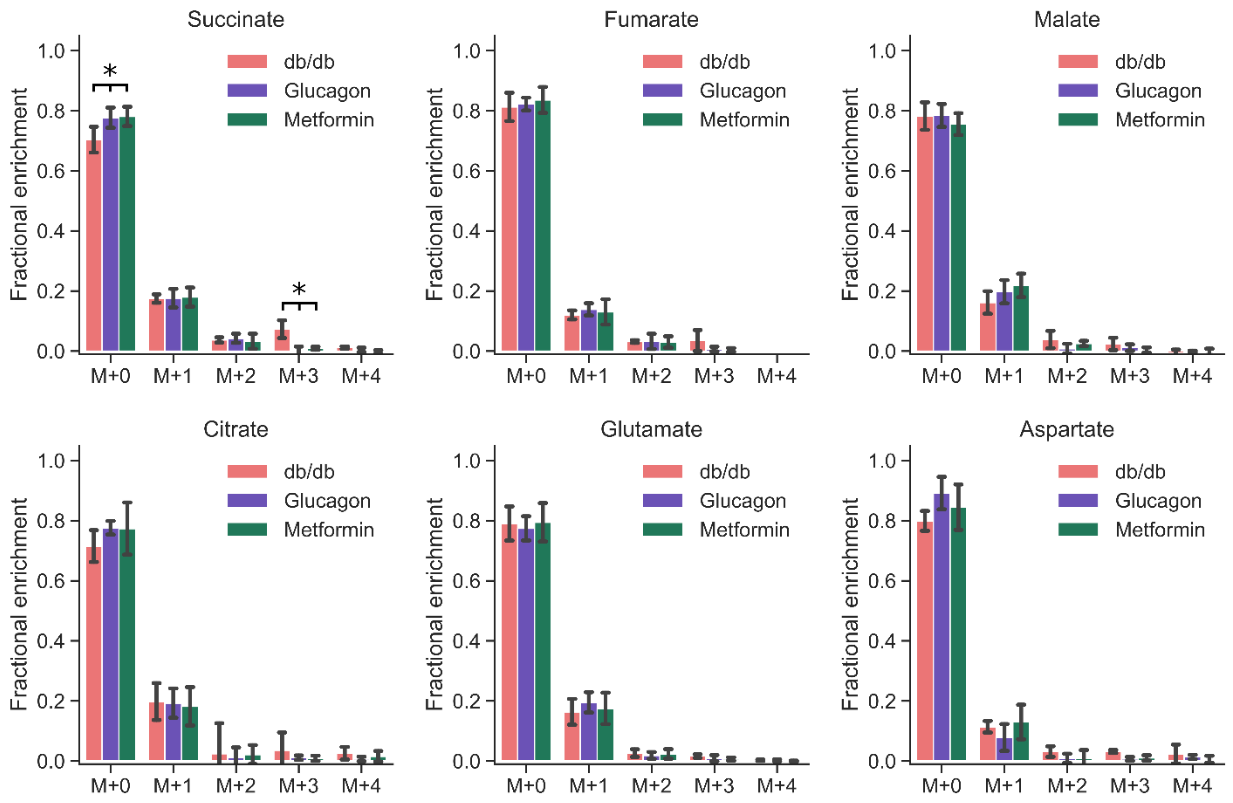

Figure S8. Fractional enrichments of citric acid cycle intermediates and closely associated metabolites extracted from the liver as estimated using GC-MS. Error bars are standard deviation. n = 6 (*db/db*), 5 (glucagon), and 6 (metformin). * denotes statistical significance ($p < 0.05$).

Table S1. Metabolites identified post HP-DHA administration from ^{13}C spectra from perfused db/db, glucagon-treated, and metformin-treated livers. "Y" indicates peaks were observed in the ^{13}C spectra. Not all metabolites were observed in every spectrum.

Metabolites	Chemical shift (ppm)	db/db & Glucagon	Metformin
[2-^{13}C] alanine	~ 51.5	Y	
[2-^{13}C] lactate	~ 69.5	Y	
[2-^{13}C] malate	~ 71.3	Y	Y
[2-^{13}C] glycerol-3-phosphate	~ 72.2	Y	Y
[2-^{13}C] glycerol	~ 73.0	Y	Y
[2-^{13}C] glyceraldehyde-3-phosphate	~ 73.8	Y	Y
[2-^{13}C] phosphoenol pyruvate	~ 150.8	Y	Y
[5-^{13}C] α - glucose-6-phosphate	~ 71.9	Y	Y
[2-^{13}C] α - glucose-6-phosphate	~ 72.6	Y	Y
[5-^{13}C] β - glucose-6-phosphate	~ 76.4	Y	
[2-^{13}C] β - glucose-6-phosphate	~ 75.4	Y	
[5-^{13}C] β – glucose	~ 76.4	Y (only db/db)	
[2-^{13}C] β – glucose	~ 75.4	Y	

Table S2. Metabolites derivatized by Methoxyamine hydrochloride and dimethyl tert butyl silyl trifluoroacetamide (MBSTFA) and their associated quantitation ions for GC-MS identification

**Metabolite**	**m/z Quantitation Ion**
Pyruvate	174-179
Lactate	261-267
Alanine	260-264
Glycine	246-250
3-hydroxybutyrate	275-279
Succinate	289-293
Fumarate	287-290
Glycerol	377-382
a-ketoglutarate	346-349
Malate	419-423
Aspartate	418-422
Glutamate	432-436
Glycerol-3-phosphate	571-577
Citrate	459-463

Table S3. Glucose fragments generated after derivatization by hydroxylamine hydrochloride and propionic anhydride and their associated quantitation ions for GC-MS identification

**Glucose Fragment**	**m/z Quantitation Ion**
Glucose C_{1-3}	215-219
Glucose C_{4-6}	258-263

References

1. Previs, S.F.; Kelley, D.E. Tracer-Based Assessments of Hepatic Anaplerotic and TCA Cycle Flux: Practicality, Stoichiometry, and Hidden Assumptions. *Am. J. Physiol. Endocrinol. Metab.* **2015**, *309*, E727–E735, doi:10.1152/ajpendo.00216.2015.
2. Satapati, S.; Kucejova, B.; Duarte, J.A.G.; Fletcher, J.A.; Reynolds, L.; Sunny, N.E.; He, T.; Nair, L.A.; Livingston, K.; Fu, X.; et al. Mitochondrial Metabolism Mediates Oxidative Stress and Inflammation in Fatty Liver. *J. Clin. Invest.* **2015**, *125*, 4447–4462, doi:10.1172/JCI82204.
